# Nanoformulation Design Including MamC-Mediated Biomimetic Nanoparticles Allows the Simultaneous Application of Targeted Drug Delivery and Magnetic Hyperthermia

**DOI:** 10.3390/polym12081832

**Published:** 2020-08-15

**Authors:** Ylenia Jabalera, Francesca Oltolina, Ana Peigneux, Alberto Sola-Leyva, Maria P. Carrasco-Jiménez, Maria Prat, Concepcion Jimenez-Lopez, Guillermo R. Iglesias

**Affiliations:** 1 Department of Microbiology, Faculty of Sciences, University of Granada, 18071 Granada, Spain; yjabalera@ugr.es (Y.J.); foltolina@ugr.es (F.O.); apn@ugr.es (A.P.); 2 Department of Biochemistry and Molecular Biology I, University of Granada, 18071 Granada, Spain; albertosola@ugr.es (A.S.-L.); mpazcj@ugr.es (M.P.C.-J.); 3 Dipartimento di Scienze della Salute, Università del Piemonte Orientale “A. Avogadro”, Via Solaroli 17, 28100 Novara, Italy; 4 Centro di Biotecnologie per la Ricerca Medica Applicata (BRMA), Via Solaroli 17, 28100 Novara, Italy; 5 Consorzio Interuniversitario per Biotecnologie (CIB), Località Padriciano 99, 34149 Area di Ricerca, Italy; 6 Consorzio Interuniversitario Nazionale per la Scienza e Tecnologia dei Materiali (INSTM), Via G. Giusti 9, 50121 Firenze, Italy; 7 Consorzio Interuniversitario di Ricerca in Chimica dei Metalli nei Sistemi Biologici (CIRCMSB), Piazza Umberto I, 1, 70121 Bari, Italy; 8 Centro Interdipartimentale di Medicina Rigenerativa (CIMeR), Via Montpellier, 1, 00133 Roma, Italy; 9 Department of Applied Physics, Faculty of Sciences, University of Granada, 18071 Granada, Spain; iglesias@ugr.es

**Keywords:** magnetic hyperthermia, magnetic nanoparticles, biomimetic magnetic nanoparticles, MamC, tumor combined dual targeting, doxorubicin

## Abstract

The design of novel nanomaterials that can be used as multifunctional platforms allowing the combination of therapies is gaining increased interest. Moreover, if this nanomaterial is intended for a targeted drug delivery, the use of several guidance methods to increase guidance efficiency is also crucial. Magnetic nanoparticles (MNPs) allow this combination of therapies and guidance strategies. In fact, MNPs can be used simultaneously as drug nanocarriers and magnetic hyperthermia agents and, moreover, they can be guided toward the target by an external magnetic field and by their functionalization with a specific probe. However, it is difficult to find a system based on MNPs that exhibits optimal conditions as a drug nanocarrier and as a magnetic hyperthermia agent. In this work, a novel nanoformulation is proposed to be used as a multifunctional platform that also allows dual complementary guidance. This nanoformulation is based on mixtures of inorganic magnetic nanoparticles (M) that have been shown to be optimal hyperthermia agents, and biomimetic magnetic nanoparticles (BM), that have been shown to be highly efficient drug nanocarriers. The presence of the magnetosome protein MamC at the surface of BM confers novel surface properties that allow for the efficient and stable functionalization of these nanoparticles without the need of further coating, with the release of the relevant molecule being pH-dependent, improved by magnetic hyperthermia. The BM are functionalized with Doxorubicin (DOXO) as a model drug and with an antibody that allows for dual guidance based on a magnetic field and on an antibody. The present study represents a proof of concept to optimize the nanoformulation composition in order to provide the best performance in terms of the magnetic hyperthermia agent and drug nanocarrier.

## 1. Introduction

The need to combine therapies to increase efficiency is gaining relevance, especially in the context of cancer [1,2]. Not only is drug combination necessary, but therapy combination too, ideally localized at the target site and possibly by using the same platform for a simultaneous application of several therapies. In the context of cancer, this is especially relevant because all the most consolidated therapies (surgery, radiotherapy, chemotherapy, and photodynamic therapy) are not fully effective, especially chemo- and radiotherapy, which provoke severe side effects due to their lack of specificity [3]. For this reason, alternative strategies are becoming attractive, and among them, magnetic hyperthermia (MH) is one of the most promising [4]. MH is the result of the application of an alternating magnetic field (AMF) to hyperthermia agents (such as iron oxide or gold nanoparticles) that, as a result, generate local heat at the tumor site. Such a local temperature increase induces the apoptosis or necrosis of tumor cells, which are more sensitive than normal healthy cells to the increased temperature of about 43 °C [5,6]. The other advantage of this approach is that MH can penetrate tissues in depth. Therefore, MH is a strong candidate to be used along with other more consolidated therapies, such as chemotherapy, to generate a synergy between the two treatments that increases the efficiency of the individual ones.

However, in order to be able to combine both therapies, a magnetic hyperthermia agent must be, at the same time, an efficient drug nanocarrier. To better perform its activity, the nanocarrier/hyperthermia agent should be specifically directed and concentrated at the target site, and this could be reached both by magnetic guidance and by a specific probe recognizing a tumor marker. Magnetic nanoparticles could be a suitable system for this goal. In fact, their magnetic properties and their ability to be functionalized by different molecules, including antibodies or enzymes, make them strong candidates to be used as a multifunctional platform for drug delivery and magnetic hyperthermia [7,8,9]. Nevertheless, it is difficult to find a magnetic nanoparticle that is efficient for both therapies, in addition to the obvious characteristics of being biocompatible and biodegradable. On one hand, the ideal magnetic nanoparticles should be superparamagnetic, i.e., behave as paramagnetic in the absence of an external magnetic field to avoid aggregation, but displaying a high magnetic susceptibility once an external magnetic field is applied. To increase the efficiency of the magnetic guidance, they should have an optimized magnetic moment per particle. These characteristics would allow the nanoparticle to respond to both a continuous gradient magnetic field used to guide them to the target, and to an alternating magnetic field, used for magnetic hyperthermia. In order to deliver nanoparticles with these characteristics, their composition, size, and shape should be optimized. For instance, magnetite nanoparticles with sizes within the range of 4–27 nm [10,11,12] and cuboctahedral versus cubic have been demonstrated to show the highest specific absorption rate (SAR) values for magnetic hyperthermia [13]. On the other hand, and to be able to combine magnetic hyperthermia with targeted drug delivery, the magnetic nanoparticles should be easily functionalized with different moieties, e.g., a drug and a probe, and to this goal, they should provide a chemical surface pattern mediating the binding for these molecules.

Inorganic magnetic nanoparticles (M) have proven to be excellent hyperthermia agents because of their small size (<30 nm), which optimizes their magnetic hyperthermia response by both magnetic Neel and Brownian relaxation, and by hysteresis losses [14,15]. However, this small size also compromises their magnetic moment per particle, and therefore the efficiency of the magnetic guidance [16]. Moreover, these MNPs need the addition of different functional groups to be functionalized with the relevant molecule, a process that shields the already not optimal magnetic moment per particle [17,18]. Therefore, inorganic MNPs could be good magnetic hyperthermia agents, but they are not optimal drug nanocarriers.

On contrary, MamC-mediated biomimetic magnetic nanoparticles (BM), which are synthetized in presence of the MamC magnetosome membrane associated protein from *Magnetococcus marinus* MC-1, display different properties compared to those of MNPs. This is a consequence of the mediation of the MamC protein in the nucleation and growth of the magnetite crystal, and of its presence of up to 5% at the surface of the nanocrystal [19,20]. Because of the unique template exerted by the protein, the nucleation of the biomimetic magnetic nanoparticles is controlled by MamC, resulting in BM that are larger than most M (average size of BM in the range 30–40 nm), and display a larger magnetic moment per particle while being superparamagnetic. Another unique feature of these BM is their surface properties. The presence of MamC attached to the surface of the BM provides functional groups to the nanoparticle without the need of additional coating and, also shifts the isoelectric point from ~7 for M to 4.4 for the BM [20]. This is crucial in making these BM optimal drug nanocarriers for two reasons: (1) at physiological pH values, BM are able to bind positively charged molecules through electrostatic interactions, which are weakened at acidic pHs (such as those found in tumor microenvironments), allowing the release of the adsorbed molecules [20,21]; (2) thanks to these electrostatic interactions, it is possible to attach to them not only the chemotherapy drug, but also to an active targeting agent, like a monoclonal antibody (mAb), as demonstrated previously by Peigneux et al. [22]. However, although these BM are optimal drug nanocarriers, they do not display as strong a magnetic hyperthermia response as that exhibited by M [23].

As a consequence of that, a previous study by Iglesias et al. [23] explored the possibility of mixing M and BM in different proportions to come up with a nanoformulation that could be used to simultaneously combine drug delivery and the hyperthermia effect. These authors show how the combination of 75% M and 25% BM had an optimal response upon the application of an alternating magnetic field and proposed this mix as the optimum nanoformulation for the potential combination of the two therapies. However, in their study, BM were not functionalized, and therefore the potential of this nanoformulation as a drug nanocarrier was anticipated, but not proven. Also, in this case, only magnetic guidance was analyzed.

In the present study, we go further with this previous idea of analyzing mixtures of M (previously referred as MNP [19]) and BM (previously referred as BMNP [19]) but, in this case, BM are functionalized with the model chemotherapy agent Doxorubicin (DOXO; here referred to as binary BM, i.e., BBM) and a monoclonal antibody (here referred as ternary BM, i.e., TBM). Therefore, the potential use of the multifunctional nanoformulation, which is susceptible of a dual guidance to the target site (mediated by the magnetic field and the monoclonal antibody) and can combine two therapeutic approaches (i.e., drug delivery and hyperthermia) is studied as a proof of concept for future in vivo applications.

## 2. Materials and Methods

### 2.1. M and BM Synthesis

MamC expression and purification were performed as previously described by Valverde-Tercedor et al. [19]. *Escherichia coli* TOP10 (Life Technologies: Invitrogen, Grand Island, NY, USA) was transformed with the plasmid pTrcHis-TOPO (Life Technologies: Invitrogen) used as a vector of the MamC protein-coding gene (Mmc1_2265) coupled to a hexahistidine tag coding sequence at its 5′ terminus. These cells were grown at 37 °C and MamC overproduction was induced with isopropyl-1-thio-β-D-galactopyranoside (IPTG). Once expressed, the purification of the protein was carried out under denaturing conditions by fast protein liquid chromatography (FPLC, GE Healthcare) by using immobilized metal affinity chromatography (IMAC, GE Healthcare, Chicago, IL, USA). Lastly, dialysis was performed for a gradual removal of urea, which allowed MamC to refold progressively, and the purity was evaluated by SDS-PAGE electrophoresis.

The synthesis of pure magnetic nanoparticles (here referred as M) was carried out at 25 °C and 1 atm total pressure using the following master solution: Fe(ClO_4_)_2_ (2.78 mM), NaHCO_3_/Na_2_CO_3_ (3.5 mM/3.5 mM), FeCl_3_ (5.56 mM), and pH 9, elaborated from oxygen-free stock concentrated solutions of the individual compounds (protocol described in [19,24]). The biomimetic nanoparticles (BM) were obtained by adding the purified MamC protein (at a final concentration of 10 µg/mL) to the solution used for M. All experiments were done under anaerobic conditions inside an anaerobic Coy chamber (96% N_2_/4% H_2,_ Coy Laboratory Products, Grass Lake, MI, USA). Samples were incubated for 30 days before the solids were magnetically decanted and washed three times with deoxygenated Milli-Q water. The magnetic particles were kept in water inside the Coy chamber until further use.

### 2.2. Functionalization of the BM

BM were functionalized with AR-3 mAbs (IgG1), which was raised against the A431 epidermoid carcinoma (ATCC^®^ CRL-1555™, Manassas, VA, USA), which was used as immunogen. The mAb was selected for its reactivity against carcinoma cells, including those of the gastro-enteric tract, and indeed it lacks reactivity with normal epithelial cells, as well as with cells of mesenchymal origin, such as fibroblasts, blood cells and sarcomas [25]. The antigen recognized by AR-3 mAb, which because of its specificity for carcinomas was called CAR-3, is a mucin-like molecule of approximately 400 kDa molecular weight and the epitope was found to be of carbohydrate nature, since it is sensitive to the metaperiodate treatment, but not to proteolytic enzymes [26]. BM were functionalized also with the chemotherapeutic agent DOXO (Sigma-Aldrich, Madrid, Spain). In both cases, functionalization was carried out as described by Iafisco et al. [27]. Briefly, 5 mg of BM were mixed with 1 mg/mL of mAb or DOXO suspended in HEPES buffer at pH 7.4 for 24 h, inside hermetically closed bottles to avoid magnetite oxidation at 25 °C, in rotation on a wheel. The DOXO-BM complexes were the so-called binary nanoparticles (BBM). Ternary nanoassemblies (mAb-DOXO-BM, here called TBM) were produced with the same protocol in two steps, coupling first the mAb and then DOXO. At the end of each incubation, particles were collected with a magnet and washed three times with HEPES buffer. Supernatants and the washings were mixed and the amounts of mAb and DOXO measured by UV–Vis spectroscopy (λ = 280 and 490 nm, respectively) were detracted from the amount initially incubated and similarly measured, to quantify the amounts of bound ligands. Functionalization efficiency was 70 ± 10% for DOXO and 40 ± 10% for AR-3 mAb corresponding to about 140 μg of DOXO and 80 μg of mAb/mg BM.

### 2.3. Nanoparticles Characterization

The morphology and size of the synthesized nanoparticles were analyzed by transmission electron microscopy (TEM Philips Model CM20, Eindhoven, The Netherlands) equipped with an energy dispersive X-ray spectrometer (EDAX). The size of the particles was analyzed by using ImageJ 1.47 software. The ζ potential, hydrodynamic radius and hysteresis cycles were measured as already described by García-Rubia et al. [20] using Malvern Zetasizer software (Malvern Instruments, Malvern, Worcestershire, UK) and a superconducting quantum interference device (SQUID) 5 T magnetometer (Quantum Design MPMS XL, San Diego, CA, USA), respectively. Powder X-ray diffraction (XRD) analysis was performed with an Xpert Pro X-ray diffractometer (PANalytical, Almelo, The Netherlands) by using the Cu Kα radiation, 20°–60° in 2θ (0.01°; 3 s). Fourier-transform infrared (FT-IR) analysis was carried out using a FTIR spectrometer (model 6600, Jasco, Tokio, Japan) equipped with an attenuated total reflection (ATR) diamond crystal window (ATR ProOne). The surface of the sample was pressed against the ATR window and infrared spectra were acquired. A total of 100 scans were collected in the wavenumber range from 4000 to 400 cm^−1^, at 2 cm^−1^ of resolution.

### 2.4. Stability Evaluation

The colloidal stability of the samples was determined optically by means of recording the sedimentation process of the different nanosystems. The time evolution of the phase separation line between the particles and medium of each sample were photographed at certain intervals. Afterwards, the height and volume of each phase were determined through image processing and analyzed. A volume of 0.5 mL 30 mg/mL of these different nanoparticles was shaken in a vortex for 1 min (time zero of the experiment) and allowed to sediment. For each experiment, the end of the sedimentation time was considered when a pellet formed at the bottom of the tube.

### 2.5. Hyperthermia Experiments

A current AC generator was used to perform the hyperthermia experiments. The setup consists of induction heating coils made by 4 turns of water-cooled copper, a power supply, and a chiller to maintain the temperature of the coil. Four frequencies, namely 143, 163, 205, and 273 kHz were selected, with a fixed magnetic field intensity of 12.5 kA/m, measured at the center of the coil, with an AC magnetic probe (NanoScience Laboratories Ltd., Newcastle, UK).

All samples were prepared in plastic 1.5 Eppendorf tubes, with a volume of 0.5 mL and a concentration of 30 mg/mL. Four types of dispersed systems were evaluated, namely those based on pure magnetic nanoparticles M, pure TBM, and mixtures containing 25% TBM + 75% M (25 TBM + 75 M) and 60% TBM + 40% M (60 TBM + 40 M).

For the measurement of hyperthermia, all samples were previously pre-thermized at 37 °C. The temperature increase as a function of time was measured with a fiber optic thermometer (Optocon AG, Dresden, Germany), and the specific absorption rate (SAR) and intrinsic loss power (ILP) of the different systems were calculated [28,29] using Equations (1) and (2):

(1) $$SAR=\left(\frac{C\cdot V_{s}}{m}\right)\frac{dT}{dt}$$

(2) $$ILP=\frac{SAR}{fH_{0}^{2}}$$

where C is the volume specific heat capacity of the sample (C_H2O_ = 4185 J/LK), vs. is the sample volume (0.5 mL in the reported experiments), and m is the mass of solids in the sample (15 mg).

### 2.6. Nanoformulation as Nanocarriers: Effect of Hyperthermia on DOXO Release

The effect of hyperthermia on the DOXO release from the nanoformulation that yielded better hyperthermia results was evaluated as described by Peigneux et al. [22]. Briefly, an alternating magnetic field (frequency = 130 kHz, H = 20 kA/m) was applied to the suspension of the 25 TBM + 75 M nanoassemblies prepared at pH 7.4 and 5.0, and DOXO release was measured at different time points up to 75 min. At specific intervals, the nanoassemblies were separated from the supernatant using a magnet and the tube was refilled with fresh buffer to continue the following time points. The magnetic field strength was controlled manually to ensure a constant temperature of 43.0 ± 0.5 °C. Identical samples kept in a thermostatic bath at 43 °C were used as control. Supernatants were measured 3 times by UV-Vis spectroscopy (λ = 490 nm).

### 2.7. Cell Cultures

The HT-29 cell line (ATCC^®^ HTB-38™, Manassas, VA, USA), derived from a human colorectal adenocarcinoma, was maintained in Minimum Essential Medium (MEM) supplemented with 10% heat-inactivated fetal bovine serum (FBS) with 2 mM l-glutamine, 1% nonessential amino acids, 100 U/mL penicillin, and 100 μg/mL streptomycin in a humid atmosphere with 5% CO_2_ as 37 °C. Cells were splitted when at 80–90% confluency.

### 2.8. Cytocompatibility and Cytotoxicity of the Nanoassemblies

HT-29 cells were seeded in 96-well plates, incubated for 24 h, and then for 72 h with 150 or 300 µg/mL of BM, M, TBM, nanoassemblies (25 BM + 75 M or 25 TBM + 75 M), and equimolar amounts of soluble DOXO coupled to the different nanoassemblies (TBM and 25 TBM + 75 M). To determine the effect of magnetic hyperthermia on cell viability, in another set of experiments, HT-29 were incubated with the maximum concentration (300 µg/mL) of M, TBM, and the nanoassembly 25 TBM + 75 M for 24 h to allow the internalization of the nanoassemblies. After the incubation time, cells were exposed to AMF for 2 h. Finally, the viability was evaluated by the MTT colorimetric assay, as already described [30]. Briefly, 10 μL of the MTT 5 mg/mL in phosphate buffered saline (PBS) solution was added to the plate, incubated at 37 °C for 2 h, and supernatants were removed. DMSO was added to dissolve the formazan crystals and the optical density was measured. The value obtained from the analyses of untreated cells run in parallel was taken as 100% viability and all the other values were normalized to the former. Experiments were performed in triplicates.

### 2.9. Statistical Analysis

Statistical analyses were performed using GraphPad Prism version 8.4.2 for Windows, GraphPad Software (GraphPad Prism, San Diego, CA, USA). For nanoparticles size characterization, size distribution curves and ANOVA statistical analyses were determined from measurements performed on 1000 particles. Averages were considered significantly different if *p* < 0.05. For in vitro biological analysis, data represent means ± SD of three independent experiments performed in triplicate, and statistical analyses were carried on using two-way ANOVA, with a Bonferroni’s post-test for grouped analysis. Statistical differences between the treatments were considered significant when *p* values were *p* ≤ 0.05 (*), *p* ≤ 0.01 (**), and *p* ≤ 0.001 (***).

## 3. Results and Discussion

### 3.1. Morphology and Particle Size

**Figure 1:**
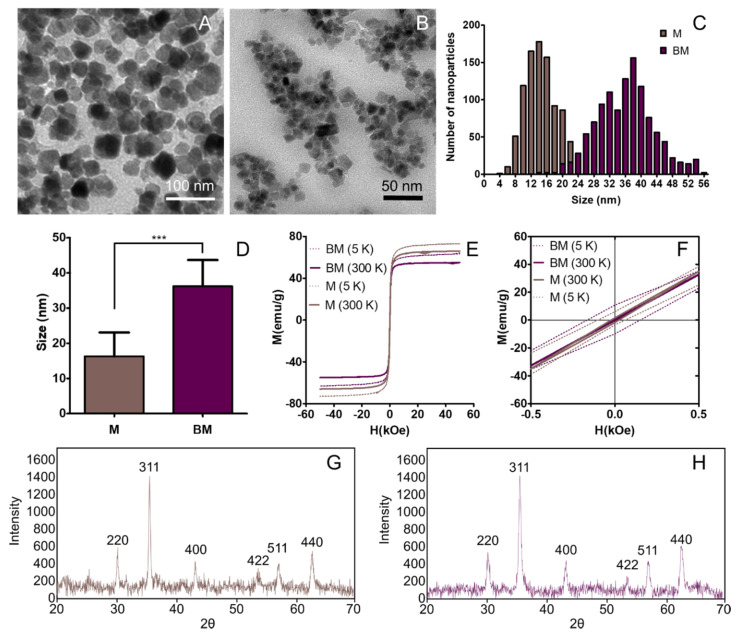
TEM images of biomimetic (**A**) and inorganic (**B**) magnetic nanoparticles. Diameter histograms of biomimetic and inorganic magnetic nanoparticles (**C**). Mean ± standard deviation of nanoparticles size (**D**) *p* < 0.001 (***). Hysteresis loop of BM and M (**E**,**F**: detail). X-ray diffraction (XRD) of M (**G**) and BM (**H**).

As shown in Figure 1A,B, M display poorly faceted crystals faces, while BM show well defined faces with different morphologies (square, rhombic, or rectangular, among others). The size of M was up to 30 nm, according to the size distribution histogram obtained from the TEM images measurements (Figure 1C,D), with an average crystal size of 15 ± 8 nm. On the contrary, BM show a size range from 10 to 60 nm, with an average size of 36 ± 8 nm. Statistical analysis (ANOVA test) reveals that the size differences between the two types of nanoparticles are statistically significant (at α < 0.05), with a probability of 7.8 × 10^−9^. The XRD patterns of M (Figure 1G) and BM (Figure 1H) demonstrate the good crystallinity of both types of magnetite nanoparticles, with the diffractions being peaks coincident with those of the reference magnetite. In fact, the diffraction peak with the highest intensity corresponds to the 311 magnetite reflection. The hysteresis loop of M and BM show the ferromagnetic character of these nanoparticles at 5 K and with no external field applied, showing remaining coercivity. However, at 300 K, the nanoparticles display zero coercivity in the absence of an external magnetic field, revealing their superparamagnetic behaviour (Figure 1E,F). The magnetization saturation of M and BM at 300 K is 63 emu/g and 55 emu/g, respectively, with BM showing less magnetization saturation because of the presence of the 5% of the MamC in the latter, as previously showed by García-Rubia et al. [20].

**Figure 2:**
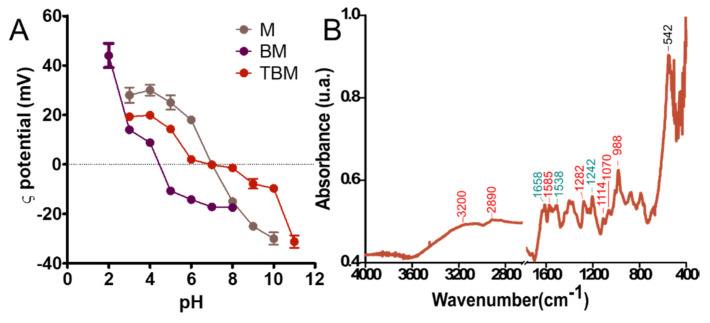
ζ potential of M, BM and TBM (**A**). ATR-FTIR spectra of TBM (**B**). The signal corresponding to different elements are marked in red (DOXO), green (AR-3 mAb) and black (magnetite).

The ζ potential values for BM and M reveal that both nanoparticles display different charge at physiological pH (Figure 2A). While M are neutral, BM are negatively charged (−20 mV), which facilities their functionalization with the relevant molecule at this physiological pH value mediated by electrostatic interactions. The BM used in this work were functionalized with DOXO and with the AR-3 mAb. This antibody, an IgG1, was chosen as a ligand model, since it reacts with a biomarker specifically expressed on different types of carcinomas, in particular those of the gastro-enteric tract, and in particular with a carbohydrate epitope present on a high molecular weight mucin [25,26]. The ability of these nanoparticles to be easily functionalized is conferred by the MamC protein. As shown in Figure 2A, at the pH value at which BM functionalization was carried out (pH value of 7.4), BM had a ζ-potential value of about −20 mV, and therefore they were negatively charged. At this pH value, the monoclonal antibody is slightly positively charged and DOXO is also positively charged due to -NH_3_^+^ groups exposed in aqueous solution, thus allowing the coupling of these two molecules to the BM based on electrostatic interactions. Identical behaviour was shown when these BM were functionalized with the monoclonal antibody DO-24 [22]. This is the great advantage of these BMs, which do not need further coating with other molecules that could alter the nanoparticles or their performance in biological systems, as previously shown by the covering of nanoparticles with AMPTES [31]. The coupling of DOXO and of the antibody to the BMs was extensively characterized by Peigneux et al. [22] (both the kinetics and thermodynamics of the coupling process and the structure and functionality of the relevant molecules after coupling), which shows that, under the conditions of the experiment, ~50% of the initial mAb in solution were first adsorbed on the BM and, after further coupling with DOXO, ~80% of the initial DOXO in solution was adsorbed at the plateau. As shown in Figure 2, the coupling of BM with DOXO and with the AR-3 mAb changes the surface properties of the nanoformulation. In fact, the ζ potential values for TBM are similar to those exposed by M nanoparticles, i.e., neutral a physiological pH. This result indicates that the negatively charged functional groups that MamC was previously exposing are now masked by DOXO and by AR-3 mAb. This functionalization is further confirmed by FT-IR analysis. The FR-IR spectra of TBM (Figure 2B) shows absorption peaks characteristics of bonds present in the different elements that constitute the TBM nanoparticles. The sample show signals at 3200, 2890, 1585, 1282, 114, 1070, and 988 cm^−1^ (marked in red), which are characteristics of the DOXO molecule [32]. On the other hand, the spectra show the relevant absorption peaks associated with the Amide I (1658 cm^−1^), II (1538 cm^−1^), and III (1242 cm^−1^) (marked in green), characteristics of mAb [33,34]. Finally, a new peak at 542 cm^−1^ characteristic of the Fe-O bond in magnetite is observed [35].

### 3.2. Magnetic Hyperthermia Responses

**Figure 3:**
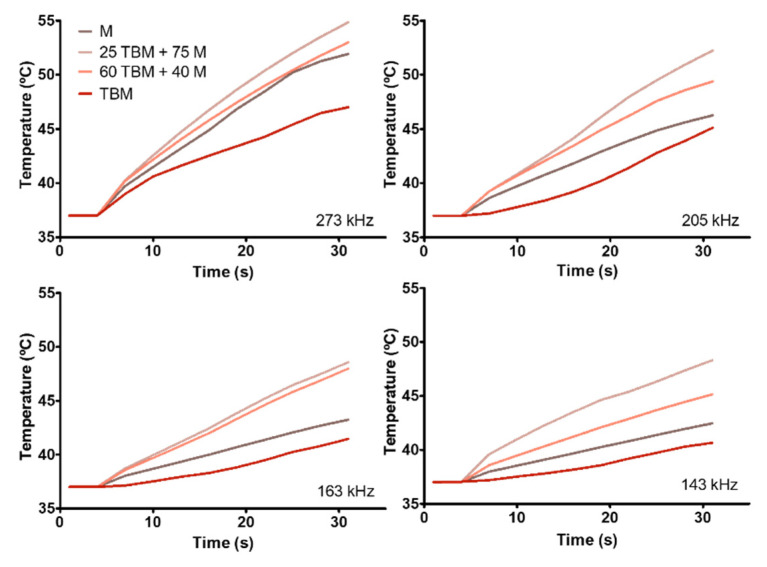
Time evolution of the temperature of the different nanoparticles suspensions, for different frequencies. Magnetic field strength: H_0_ = 12.5 kA/m. Sample volume 0.5 mL; particle concentration: 30 mg/mL.

In terms of how these M and TBM behave upon the application of an alternating magnetic field, Figure 3 shows that they both are able to increase the temperature in a frequency dependent way, the higher increase corresponding to the higher frequencies. Comparing the data of the TBM from the present paper to those of BM already published by Peigneux et al. [22] and Iglesias et al., [23], it can be concluded that the presence of the molecules adsorbed to the BM does not interfere significantly with the ability of TBM to respond to an alternating magnetic field. However, data in Figure 3 and Table 1 show that M display a better behavior compared to BM and TBM in producing hyperthermia [23], which might be depended on their higher relation in magnetic/non-magnetic material compared to BM and TBM, as also observed for other encapsulated magnetic nanoparticles [6,36].

**Figure 4:**
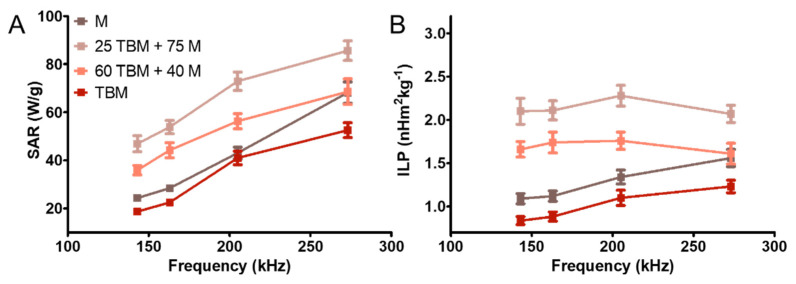
Frequency dependence of SAR (**A**) and ILP (**B**) for the different nanoparticles suspension. Magnetic field strength: 12.5 kA/m; particle concentration: 30 mg/mL.

Interestingly, when these two systems are combined, the temperature increase is higher compared to that of the endmembers, being the nanoformulation 25 TBM + 75 M the one that induce the higher temperature increase. In all cases, the temperature needed to induce apoptosis in tumor cells (42–46 °C [7,37]) is reached in a few seconds (~30 s) under an alternating magnetic field of 12.5 kA/m. The SAR and ILP values are represented in Figure 4 (Table 1) and are in agreement with the results obtained in [23].

### 3.3. Colloidal Stability

**Figure 5:**
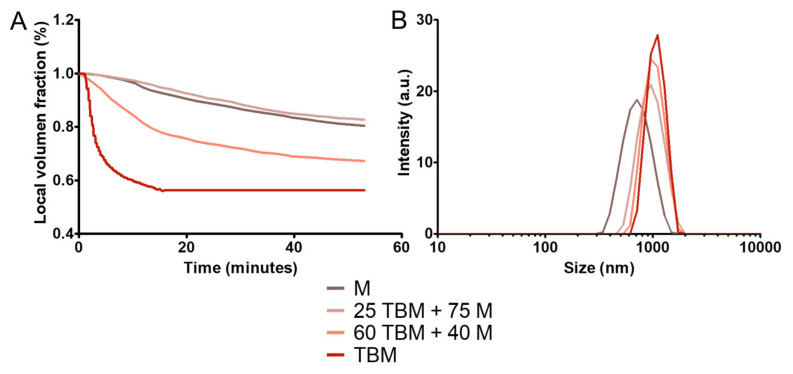
Colloidal stability of M, 25 TBM + 75 M and 60 TBM + 40 M. Height normalized to its initial volume value as a function of time (**A**). Hydrodynamic size in intensity distribution for the different formulations (**B**).

Colloidal stability against agglomeration and sedimentation is one of the main requirements for biomedical applications of nanoparticles, this stability being influenced by different factors such as volume fraction, size distribution and temperature [38]. In general, to avoid aggregation and precipitation, the nanoparticles need to be further coated with a long chain polymer. This coating is not necessary in our BM nanoparticles due to the presence of MamC. However, the larger sizes of BM and TBM compared to that of M could affect their colloidal stability. The colloidal stability analysis shown in Figure 5A (Figure S1) reveals that the 25 TBM + 75 M sample shows the highest stability, similar to that exhibited by M, followed in decreasing order by 60 TBM + 40 M and TBM samples. This fact could probably be the result of a stabilizing effect mediated by the interaction of the smaller M size with the TBM coating [23]. Smaller M nanoparticles could intercalate within the TBM nanoassemblies and potentiate the electrostatic repulsion being both negatively charged at physiological pH values (pH = 7.4, M −7.5 mV and TBM −1.4 mV). The smaller the amount of M, the lower this electrostatic repulsion is and the slight charge of TBM at physiological pH values may not be enough to force electrostatic repulsion between nanoassemblies, with TBM thus being prone to agglomeration. These results are in agreement with the hydrodynamic radius data (Figure 5B). Dynamic light scattering (DLS) analysis show that the hydrodynamic diameters of the M, 25 TBM + 75 M, 60 TBM + 40 M and TBM are of 700 ± 100 nm, 900 ± 100 nm, 1200 ± 100 nm, and 1300 ± 100 nm, respectively. The polydispersity index (PDI) of these samples are 0.249 ± 0.004, 0.214 ± 0.008, 0.283 ± 0.001, and 0.285 ± 0.002, respectively.

Previous studies have demonstrated that M display better magnetic hyperthermia response than BM and TBM (as shown in Figure 3 and Table 1), due to the difficulty for rotation conditioned by the MamC protein coating and their larger size [20,23,39]. However, the combination of M and TBM yielded the highest values for the hyperthermia analyses. In fact, the maximum SAR and ILP values were obtained by 25 TBM + 75 M system, followed by 60 TBM + 40 M, M and TBM (Table 1, Figure 4). These increased SAR and ILP values could be explained by the higher colloidal stability of the 25 TBM + 75 M system compared to that of the 60 TBM + 40 M (Figure 5), since aggregation negatively affects the rotation of the nanoparticles under the influence of the AMF, and thus the temperature rise. Moreover, suspensions of nanoparticles with broad size distributions have been proposed to yield an optimized magnetic hyperthermia response, since, in this case, the probability that a percentage of the nanoparticles in the system are always rotating in response to a given AMF is maximized [28]. This would apply to our mixed system, which combines magnetic nanoparticles with sizes up to 70 nm, showing a broader size distribution compared to that of the end members (up to 30 nm in M and up to 70 nm in TBM; Figure 1). In consequence, a higher percentage of the nanoparticles are likely to be affected by the influence of the magnetic field in the mixture compared to that in the end members, thus resulting in a more efficient temperature rise produced by the former.

### 3.4. Nanoformulation as DOXO Nanocarrier. Synergy of Magnetic Hyperthermia and of pH Decrease in the Drug Release

**Figure 6:**
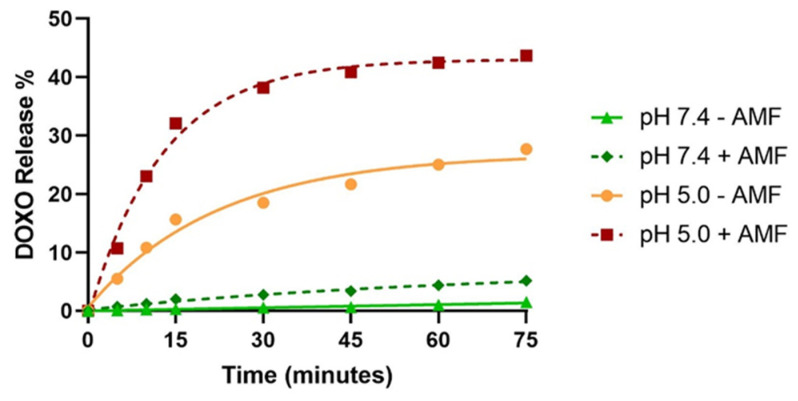
DOXO release percentage as a function of time measured with and without (AMF) at different pHs (7.4 and 5.0). The vertical error bars are smaller than the symbol.

When the mixture of 25 TBM + 75 M was incubated up to 75 min at a pH value of 7.4 (physiological pH value), less than 2% of the adsorbed DOXO was released from TBM (Figure 6), thus indicating the stability of the nanoformulation at physiological pH values. However, when the nanoformulation was incubated in an acidic environment mimicking the one in the endosomal/lysosomal compartment (pH 5.0) the release of DOXO increased to more than 25%, and this value was further increased to 44% when an AMF treatment (H = 20 kA/m, *f* = 130 kHz) for 30 min was applied. This synergy between acidic pH and AMF on the release of the DOXO coupled to MamC-mediated BM was previously reported by Peigneux et al. [22]. These authors showed that the electrostatic bond, which at higher pH values kept DOXO bound to the surface of the nanoparticles, weakens as BM approach their iep (4.4), thus triggering DOXO release. Following application of the AMF, the kinetics of DOXO release is probably faster because of the heating of the BM surface [22]. However, this heating is not enough to break the electrostatic bond that keeps DOXO attached to the nanoparticle at physiological pH values (DOXO release increases from 2% to 5% after the application of AMF), while, at acidic pH values, it is enough to break the electrostatic bond once the BM surface is no longer so negative. The enhancing effect of drug release induced by magnetic hyperthermia was pointed out by other authors, using different drugs and magnetic nanocarriers [8,40,41]. This synergy further confirms our hypothesis that the effectiveness of the treatment could be increased by combining the two therapies of drug delivery and hyperthermia by using this multifunctional nanoformulation, which moreover allows a dual tumor targeting (magnetic field and monoclonal antibody).

### 3.5. Cytotoxicity Evaluation

**Figure 7:**
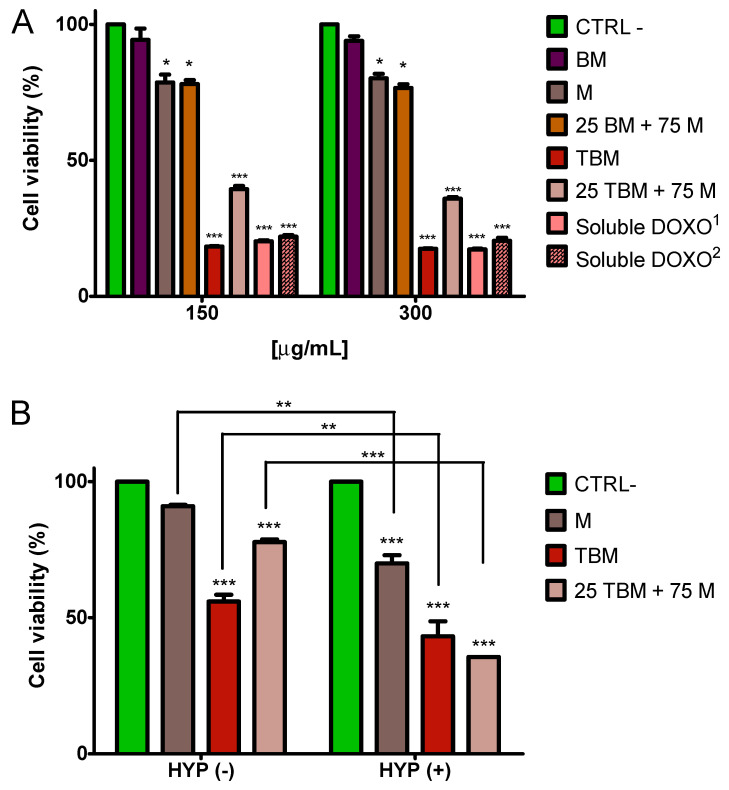
Cytotoxicity of the differentially functionalized nanoassemblies on HT-29 cells measured in MTT assay after 72 h without AMF (**A**) and during 24 h with AMF (**B**). Soluble DOXO^1^ (36 μg/mL) corresponds to the equimolar amount of drug loaded on TBM sample. Soluble DOXO^2^ (9 μg/mL) corresponds to the equimolar amount of drug loaded on 25 TBM + 75 M sample. Control cells (CTRL-) not incubated with nanoparticles or DOXO were taken as reference value (100%) of viable cells to which refer the values of treated cells. *p* ≤ 0.05 (*), *p* ≤ 0.01 (**), *p* ≤ 0.001 (***).

The toxic activity of the different nanoformulations (25 BM + 75 M and 25 TBM + 75 M), and their respective components, were assessed in an MTT assay carried out on the colon carcinoma HT-29 cell line, which express the AR-3 mAb-defined biomarker, after 72 h of treatment. Soluble DOXO was used at the same concentrations as that contained in the nanoformulations, as positive control. Figure 7A shows the cell viability compared to that of the untreated controls (CTRL-). As previously shown by our groups [20,22,30,42], BM are cytocompatible. Indeed, both M and DOXO-free 25 BM + 75 M nanoformulations display low cytotoxicity, and in all cases cell viability is above 80%, which is considered the cut-off indicated by ISO 10993–5:2009 [43]. By contrast, TBM and the 25 TBM + 75 M mixture display a significant toxicity. In particular, for higher doses of DOXO (DOXO^1^), as in the case of TBM, no differences were observed in respect to soluble DOXO, while for lower doses of DOXO (around ¼), as in the case of 25 TBM + 75 M, the soluble DOXO (DOXO^2^) was more efficient than the DOXO coupled to TBM. Similar findings have already been reported, both for MNPs [22,44] and for other functionalized inorganic NPs [27,45], and are explained by the fact that soluble DOXO can more easily diffuse through the cell membrane.

When cells were exposed to AMF for 2 h after 24 h of incubation with M, TBM, and DOXO nanoassemblies (25 TBM + 75 M), it was observed that, under all experimental conditions, the application of AMF significantly reduced cell viability in respect to control cells incubated in the absence of magnetic nanoparticles. Moreover, despite the fact that TBM has a higher DOXO load than the 25 TBM + 75 M nanoformulation, the decrease in cell viability was more evident when cells were incubated with the latter formulation, an effect that may be due to the higher hyperthermia caused by 25 TBM + 75 M nanoformulation (Figure 7B). These data suggest that a significant level of cytotoxicity can be also reached or even improved with a lower amount of drug by using a mixture of magnetic nanoparticles that increases the hyperthermia response.

## 4. Conclusions

Results from the present study show that the nanoformulation here proposed, integrated by a combination of two different types of magnetic nanoparticles, one inorganic with an average size of ~15 nm, and the other biomimetic (mediated by the magnetosome associated protein MamC) with an average size of ~36 nm, can be used as a multifunctional platform for a combined therapy. Indeed, the inorganic nanoparticles show good potential as magnetic hyperthermia agents, while the biomimetic nanoparticles are better suitable as nanocarrier, being able to be functionalized with a drug and a targeting probe by electrostatic interaction, being the release of the drug pH dependent. Both particles are superparamagnetic and are able to respond to a continuous gradient field used for guiding them at the target site. The mixture of the two types of nanoparticles overcome the already good potential demonstrated for the pure end members, in terms of colloidal stability and magnetic hyperthermia response. Moreover, it allows a synergy between drug delivery and magnetic hyperthermia that increases the release of the relevant drug at acidic conditions mimicking tumor sites. Thus, this multifunctional combined platform could reach the target site by the application of dual guidance (external magnetic field and active targeting antibody), and could act as an anti-tumor agent in a bimodal way: by hyperthermia upon the application of an alternated magnetic field and by carrying and releasing the drug in pH- and temperature-dependent manners. Data from the present study may help in optimizing new treatments in which cytotoxicity is raised not by increasing the dose of the chemotherapeutic agent, but by the simultaneous use of magnetic hyperthermia.

## Figures and Tables

**Table 1:** Summary of SAR and ILP calculations at different frequencies with a fixed magnetic field intensity of 12.5 kA/m.

System	Frequency *f* [kHz]	SAR [W/g]	Slope dT/dt [°C/s]	ILP [nHm^2^kg^−1^]
M	273 ± 5	68 ± 4	0.50 ± 0.02	1.6 ± 0.1
	205 ± 5	47 ± 2	0.345 ± 0.009	1.34 ± 0.08
	163 ± 5	28 ± 1	0.202 ± 0.005	1.12 ± 0.06
	143 ± 5	24 ± 1	0.175 ± 0.004	1.09 ± 0.06
25 TBM + 75 M	273 ± 5	86 ± 4	0.58 ± 0.02	2.1 ± 0.1
	205 ± 5	73 ± 4	0.50 ± 0.01	2.3 ± 0.1
	163 ± 5	54 ± 3	0.379 ± 0.009	2.1 ± 0.1
	143 ± 5	47 ± 3	0.36 ± 0.02	2.1 ± 0.2
60 TBM + 40 M	273 ± 5	69 ± 5	0.51 ± 0.02	1.6 ± 0.1
	205 ± 5	56 ± 3	0.41 ± 0.01	1.8 ± 0.1
	163 ± 5	44 ± 3	0.350 ± 0.009	1.7 ± 0.1
	143 ± 5	36 ± 2	0.262 ± 0.007	1.66 ± 0.09
TBM	273 ± 5	53 ± 3	0.37 ± 0.02	1.23 ± 0.07
	205 ± 5	41 ± 2	0.27 ± 0.02	1.17 ± 0.08
	163 ± 5	22 ± 1	0.162 ± 0.009	0.88 ± 0.05
	143 ± 5	19 ± 1	0.134 ± 0.007	0.84 ± 0.04

## Supplementary Materials

The following are available online at https://www.mdpi.com/2073-4360/12/8/1832/s1, Figure S1. Colloidal stability video of the samples measured using photograms of the sedimentation time evolution.
