# Supplementary material for: Nanoformulation Design Including MamC-Mediated Biomimetic Nanoparticles Allows the Simultaneous Application of Targeted Drug Delivery and Magnetic Hyperthermia
Source: Polymers (Basel). 2020 Aug 15;12(8):1832. doi: 10.3390/polym12081832 (PMC7465699; doi:10.3390/polym12081832)
Supplement: Supplementary file 1 [file polymers-12-01832-s001.zip › Supplementary Information.pdf]

Supporting Information

Nanoformulation Design Including MamC-Mediated Biomimetic Nanoparticles Allows the Simultaneous Application of Targeted Drug Delivery and Magnetic Hyperthermia

Ylenia Jabalera[1,†], Francesca Oltolina[1,†], Ana Peigneux[1], Alberto Sola-Leyva[2], Maria P. Carrasco-Jiménez[2], Maria Prat[3,4,5,6,7,8,*], Concepcion Jimenez-Lopez[1,*] and Guillermo R. Iglesias[9]

[1] Department of Microbiology, Faculty of Sciences, University of Granada, 18071 Granada, Spain; yjabalera@ugr.es (Y.J.); foltolina@ugr.es (F.O.); apn@ugr.es (A.P.); cjl@ugr.es (C.J.-L.);
[2] Department of Biochemistry and Molecular Biology I, University of Granada, 18071 Granada, Spain; albertosola@ugr.es (A. S.-L.); mpazcj@ugr.es (M.P. C-J).
[3] Dipartimento di Scienze della Salute, Università del Piemonte Orientale "A. Avogadro", Via Solaroli 17, 28100 Novara, Italy; maria.prat@med.uniupo.it (M.P.);
[4] Centro di Biotecnologie per la Ricerca Medica Applicata (BRMA), Via Solaroli 17, 28100 Novara, Italy
[5] Consorzio Interuniversitario per Biotecnologie (CIB), Località Padriciano 99, 34149 Area di Ricerca, Italy
[6] Consorzio Interuniversitario Nazionale per la Scienza e Tecnologia dei Materiali (INSTM), Via G. Giusti 9, 50121 Firenze, Italy
[7] Consorzio Interuniversitario di Ricerca in Chimica dei Metalli nei Sistemi Biologici (CIRCMSB), Piazza Umberto I, 1, 70121 Bari, Italy
[8] Centro Interdipartimentale di Medicina Rigenerativa (CIMeR), Via Montpellier, 1, 00133 Roma, Italy
[9] Department of Applied Physics, Faculty of Sciences, University of Granada, 18071 Granada, Spain; iglesias@ugr.es (G.R.I.).
* Correspondence: cjl@ugr.es (C.J.-L.) (+34-958-249-833); maria.prat@med.uniupo.it (M.P.) (+39-0321-660-662)

Figure S1. Colloidal stability of the samples measured using photograms of the sedimentation time evolution**.**

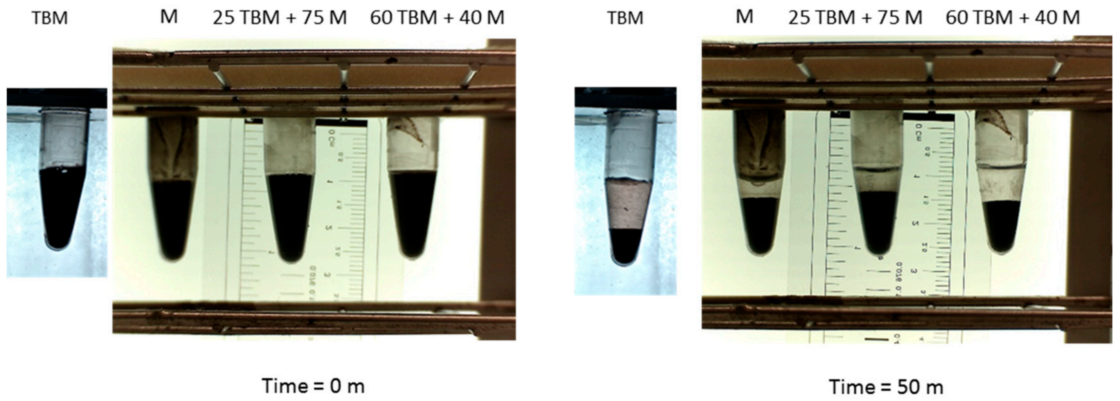

Video x 150 (1 frame = 150 s)

Total video time 20s
